# Engineering Catalytic Nanozymes for Antimicrobial Food Systems: Structure–Activity Relationships, Safe-by-Design Principles, and Industrial Translation

**DOI:** 10.3390/nano16140887

**Published:** 2026-07-19

**Authors:** Huy Loc Nguyen, Thi Bich Ngoc Nguyen

**Affiliations:** 1 Department of Engineering and Technology, Van Hien University, Ho Chi Minh City 72419, Vietnam; 2 Department of Water Management and Hydrological Sciences, Texas A&M University, College Station, TX 77843, USA; nguyent0313@tamu.edu

**Keywords:** antimicrobial nanomaterials, biofilm control, catalytic nanozymes, food safety, reactive oxygen species, safe-by-design, structure–activity relationships

## Abstract

Catalytic nanozymes have emerged as a versatile class of engineered nanomaterials that combine enzyme-like catalytic activity with exceptional physicochemical stability, tunable composition, and scalable fabrication, offering significant advantages over natural enzymes for antimicrobial applications in food systems. Recent advances in materials engineering have enabled the development of nanozymes with enhanced catalytic efficiency, broad-spectrum antimicrobial activity, and improved resistance to harsh food-processing environments. Nevertheless, current research remains fragmented across diverse material platforms and application scenarios, while a comprehensive understanding of how engineering strategies govern catalytic performance, antimicrobial efficacy, and translational potential is still lacking. This review provides a critical and systematic analysis of catalytic nanozymes for antimicrobial food systems from a structure–activity relationship perspective. Emphasis is placed on the engineering principles that regulate enzyme-mimicking activities, including compositional tuning, crystal phase and facet engineering, defect creation, heterostructure construction, pore architecture, surface functionalization, and single-atom engineering. The relationships between these structural features and catalytic mechanisms, including peroxidase-, oxidase-, catalase-, and multi-enzyme-like activities, are discussed in relation to the generation of reactive oxygen species, membrane disruption, extracellular polymeric substance degradation, biofilm eradication, and pathogen inactivation. Representative applications in food-contact surface decontamination, antimicrobial packaging, fresh produce preservation, and intelligent food processing are critically evaluated using recent experimental evidence. Beyond antimicrobial performance, this review introduces a safe-by-design framework that integrates material engineering with toxicological assessment, nanoparticle migration, environmental fate, regulatory considerations, and scalable manufacturing. Emerging opportunities for artificial intelligence-assisted nanozyme design, high-throughput materials discovery, and data-driven optimization are also discussed as transformative approaches for accelerating industrial translation. By integrating materials science, catalytic mechanisms, food microbiology, and safety assessment, this review establishes a comprehensive framework for the rational development of next-generation catalytic nanozymes toward sustainable, effective, and industrially applicable antimicrobial food systems.

## 1. Introduction

Foodborne pathogens remain a persistent threat to public health and food-system resilience. Global burden assessments have shown that contaminated foods contribute substantially to morbidity, mortality, and disability-adjusted life years worldwide, with bacterial pathogens such as *Salmonella enterica*, pathogenic *Escherichia coli*, *Listeria monocytogenes*, *Campylobacter* spp., and *Staphylococcus aureus* representing major etiological agents of foodborne illness [1,2]. In addition to acute infection, the control of foodborne bacteria is complicated by their ability to persist on food-contact surfaces, processing equipment, fresh produce, meat, dairy products, and packaging interfaces. Once attached to surfaces, bacterial cells can develop structured biofilms embedded within extracellular polymeric substances (EPS), which restrict sanitizer penetration, reduce antimicrobial susceptibility, and facilitate long-term contamination in food-processing environments [3]. These challenges are especially relevant for ready-to-eat foods, minimally processed fresh produce, refrigerated foods, and high-moisture food matrices, where severe thermal processing is either undesirable or incompatible with product quality.

Conventional antimicrobial interventions, including chlorine-based sanitizers, organic acids, thermal treatment, ultraviolet irradiation, ozone, and chemical preservatives, remain central to food safety management [4,5]. However, their performance can be limited by matrix complexity, incomplete biofilm removal, potential formation of undesirable residues or by-products, corrosive effects on equipment, and adverse effects on sensory or nutritional quality. Moreover, many conventional antimicrobial assessments rely heavily on planktonic bacterial inactivation or plate-count reduction, which may not fully represent antimicrobial performance against attached cells, mature biofilms, or microorganisms embedded within organic matter. Therefore, next-generation antimicrobial strategies for food systems, such as nanozyme-based antimicrobials, metal–organic framework (MOF)-based systems, or phage-derived endolysins, are applied and should ideally combine high microbial inactivation efficiency, biofilm-disruptive capability, environmental stability, low residue risk, compatibility with food matrices, and scalability for industrial implementation [6,7].

Catalytic nanozymes have emerged as a promising class of engineered nanomaterials that mimic natural enzyme activities while offering superior physicochemical stability, tunable structures, and greater resistance to harsh processing conditions. Since the discovery that magnetite nanoparticles possess intrinsic peroxidase-like activity [8], diverse nanozyme platforms have been developed, including metal oxides, noble metals, carbon-based nanomaterials, metal–organic frameworks (MOFs), covalent organic frameworks, MXenes, single-atom catalysts, and hybrid nanocomposites. These materials can mimic peroxidase-, oxidase-, catalase-, superoxide dismutase-, haloperoxidase-, and multi-enzyme-like activities, enabling catalytic regulation of reactive oxygen species (ROS), electron transfer, redox cycling, and substrate oxidation [9,10]. Unlike conventional antimicrobial nanoparticles that primarily depend on ion release, passive membrane contact, or photodynamic effects, nanozymes can actively catalyze antimicrobial reactions under specific microenvironmental conditions [11]. This catalytic feature makes them particularly attractive for food systems, where pathogen inactivation, biofilm disruption, active packaging, and intelligent preservation may require responsive and sustained antimicrobial performance.

The antimicrobial activity of nanozymes is closely associated with their catalytic ability to generate or regulate ROS, including hydroxyl radicals, superoxide anions, singlet oxygen, and hypohalous species [12]. These reactive intermediates can damage bacterial membranes, oxidize proteins and lipids, disrupt DNA, impair energy metabolism, and degrade EPS components within biofilms. For example, vanadium pentoxide nanowires were shown to mimic vanadium haloperoxidases and suppress biofilm formation through catalytic halogenation reactions [13]. Iron oxide nanozymes, including ferumoxytol-based systems, have demonstrated the ability to activate hydrogen peroxide under acidic biofilm microenvironments, resulting in bacterial killing and EPS degradation [14]. More recently, surface-bound ROS-generating nanozymes were reported to selectively kill bacteria over mammalian cells, suggesting that spatial confinement of ROS at the nanozyme surface may improve antimicrobial selectivity and reduce off-target toxicity [15]. These findings indicate that antimicrobial efficacy is not governed solely by ROS quantity, but also by ROS localization, lifetime, diffusion distance, catalytic microenvironment, and nanozyme–cell interfacial interactions.

A key challenge in advancing catalytic nanozymes for antimicrobial food applications is the incomplete understanding of structure–activity relationships (SARs). Nanozyme activity is influenced by composition, oxidation state, particle size, morphology, crystal phase, exposed facets, defects, porosity, surface charge, ligand chemistry, and interfacial electronic structure. For instance, pH-switchable enzyme-like activities have been reported for noble-metal nanoparticles, demonstrating that catalytic behavior can shift depending on reaction conditions and surface chemistry [16]. Single-atom nanozymes further provide atomically dispersed active sites with high catalytic efficiency and tunable coordination environments. Copper single-atom catalysts on nitrogen-doped porous carbon have been developed for photothermal-catalytic antibacterial therapy, while Fe–N–C and Mn single-atom nanozymes have shown enhanced peroxidase-like or Fenton-like activity for bacterial inactivation [17,18]. Similarly, heterostructure nanozymes can create interfacial charge redistribution, accelerate electron transfer, and enhance ROS generation, as demonstrated in photo-enhanced and hyperthermia-amplified antibacterial systems [19]. These studies collectively suggest that rational nanozyme engineering can transform antimicrobial design from empirical material screening toward mechanism-guided optimization.

For food systems, catalytic nanozymes are increasingly being explored beyond conventional antibacterial testing. Nanozyme-mediated signal amplification has enabled sensitive detection of foodborne pathogens in complex matrices. A Pt nanoparticle-modified Zr-MOF nanozyme was integrated into a microfluidic immunosensor for *E. coli* O157 detection in water, milk, and cabbage samples, achieving rapid and sensitive analysis within a practical testing format [20]. Bifunctional nanozyme-mediated catalytic amplification combined with SERS has also been used for ultrasensitive detection of pathogens in milk samples [21]. Prussian blue nanoparticles have served as peroxidase-mimicking labels for simultaneous immunochromatographic detection of *Salmonella typhimurium* and *Listeria monocytogenes* in milk [22]. In parallel, multifunctional nanozyme platforms have been developed for combined detection and on-demand disinfection of *L. monocytogenes*, indicating an emerging detect-and-eliminate direction for food safety applications [23]. These advances are important because industrial food safety requires not only pathogen inactivation but also rapid monitoring, trace-level detection, and compatibility with real food matrices.

Nanozymes are also beginning to enter active food preservation and packaging-related applications. Chitosan composite Ce-MOF nanozyme coatings have been reported for fruit preservation, showing oxidase- and apyrase-like activities, antibacterial effects against *E. coli* and *S. aureus*, and improved storage performance of strawberries and bananas [24]. More recently, H_2_O_2_-activatable peptide nanozyme–alginate dual-network hydrogel coatings were designed to reconcile antibacterial activity with moisture retention during strawberry preservation [25]. These studies suggest that nanozymes may be engineered as active coatings, sprayable preservatives, hydrogel networks, packaging additives, or surface treatments capable of providing controlled catalytic antimicrobial activity. However, translating such systems from laboratory-scale demonstrations to industrial food applications requires careful consideration of food matrix interference, catalytic durability, sensory impact, material stability, cost, processing compatibility, and regulatory acceptance.

Despite the rapid growth of nanozyme research, several gaps limit their responsible application in antimicrobial food systems. First, many studies report antibacterial activity without sufficiently connecting material structure to catalytic mechanism and biological outcome. Second, antimicrobial performance is often evaluated under simplified aqueous conditions rather than in realistic food matrices containing proteins, lipids, carbohydrates, salts, phenolics, and organic acids, all of which may affect ROS generation, nanozyme aggregation, or surface fouling. Third, food-contact applications require more rigorous safety evaluation than many biomedical proof-of-concept studies because consumer exposure may occur through repeated contact with food, potential nanoparticle migration, ion release, and environmental discharge. Nanoparticle migration remains an important consideration for food-contact nanozymes because consumer exposure depends on material composition, processing conditions, and food characteristics [26,27]. Therefore, antimicrobial efficacy alone is insufficient for industrial translation; nanozyme development must be integrated with safe-by-design principles.

Safe-by-design nanozyme engineering requires that antimicrobial performance, catalytic selectivity, toxicity, migration, environmental fate, and end-of-life behavior be considered from the earliest stages of material design. For food applications, this includes minimizing unnecessary nanoparticle release, selecting food-compatible components, controlling metal ion leaching, reducing persistence in biological and environmental systems, and validating performance under realistic processing and storage conditions [28]. Recent safe-and-sustainable-by-design approaches for nanomaterial-containing food packaging demonstrate the value of integrating functionality, exposure assessment, and sustainability considerations into material development [29]. Applying this logic to catalytic nanozymes could help shift the field from performance-driven discovery toward responsible innovation.

This review focuses on engineering catalytic nanozymes for antimicrobial food systems, with emphasis on structure–activity relationships, safe-by-design principles, and industrial translation. Rather than simply categorizing nanozymes by material type, this review examines how rational engineering strategies, including compositional tuning, defect creation, crystal phase and facet regulation, heterostructure construction, pore architecture, surface functionalization, and single-atom engineering, govern catalytic activity and antimicrobial performance. The review further discusses nanozyme-mediated mechanisms of pathogen inactivation, biofilm disruption, foodborne pathogen detection, food-contact surface decontamination, active packaging, and postharvest preservation. Finally, translational challenges related to food matrix effects, biosafety, nanoparticle migration, regulatory requirements, scalability, life-cycle considerations, and data-driven nanozyme design are critically evaluated. By integrating materials science, catalytic mechanisms, food microbiology, and safety assessment, this review aims to provide a framework for the rational and responsible development of next-generation catalytic nanozymes for sustainable antimicrobial food systems.

## 2. Methodologies

This review was prepared through a structured literature search and critical analysis of published studies related to catalytic nanozymes, antimicrobial nanomaterials, foodborne pathogen control, biofilm eradication, food preservation, food-contact surfaces, and safe-by-design nanotechnology. The literature was collected from major scientific databases, including Web of Science, Scopus, PubMed, ScienceDirect, SpringerLink, ACS Publications, Wiley Online Library, and Google Scholar. The search focused primarily on peer-reviewed research articles published between 2010 and 2026, with particular attention to recent studies published after 2020. Review articles were used only when necessary to provide a general background or to identify additional primary research sources.

The search strategy combined keywords such as “*nanozyme*”, “*catalytic nanomaterials*”, “*enzyme-mimicking nanoparticles*”, “*antimicrobial nanozymes*”, “*foodborne pathogens*”, “*biofilm eradication*”, “*reactive oxygen species*”, “*food preservation*”, “*food-contact surfaces*”, “*active food packaging*”, “*structure–activity relationships*”, “*safe-by-design*”, “*nanoparticle migration*”, and “*industrial translation*”. Studies were included when they reported original experimental data on nanozyme synthesis, catalytic activity, antimicrobial performance, pathogen detection, biofilm control, food preservation, or safety-related evaluation. Articles focusing only on biomedical therapy without clear relevance to antimicrobial mechanisms, foodborne pathogens, or translational material design were considered only when they provided important mechanistic insights.

Relevant information was extracted and organized according to nanozyme type, material composition, engineering strategy, enzyme-mimicking activity, catalytic mechanism, antimicrobial target, food-related application, safety considerations, and translational relevance. Focus was placed on studies that connected structural properties, such as size, morphology, crystal phase, defects, surface chemistry, heterostructures, porosity, or single-atom sites, with catalytic and antimicrobial outcomes. The selected literature was then critically analyzed to identify major advances, structure–activity relationships, knowledge gaps, and future research directions for the safe and scalable use of catalytic nanozymes in antimicrobial food systems.

## 3. Catalytic Nanozyme Platforms and Engineering Logic for Antimicrobial Food Systems

### 3.1. Representative Nanozyme Platforms and Enzyme-Mimicking Activities

Catalytic nanozymes constitute a broad family of nanomaterials capable of mimicking natural enzyme activities while retaining the structural tunability and environmental robustness of engineered nanomaterials. The field was strongly accelerated by the discovery that Fe_3_O_4_ nanoparticles possess intrinsic peroxidase-like activity, allowing them to catalyze chromogenic substrate oxidation in the presence of hydrogen peroxide [30]. This finding established an important conceptual foundation: catalytic behavior can arise from nanoscale surface chemistry rather than from biological macromolecular structures. Since then, a wide range of metal oxides, noble metals, carbon-based materials, metal–organic frameworks (MOFs), and hybrid nanocomposites have been developed as enzyme-mimicking antimicrobial platforms [31].

Among metal oxide nanozymes, Fe_3_O_4_, Co_3_O_4_, CeO_2_, MnO_2_, and V_2_O_5_ have received substantial attention because their variable oxidation states can mediate redox cycling and reactive oxygen species (ROS) regulation. Co_3_O_4_ nanoparticles, for example, exhibit both peroxidase- and catalase-like activities, highlighting the possibility of multi-enzyme behavior within a single inorganic platform [32]. Noble-metal nanozymes, including Au, Ag, Pt, and Pd nanoparticles, can show pH-dependent peroxidase- or catalase-like activities, indicating that catalytic function is strongly controlled by reaction microenvironment [10]. V_2_O_5_ nanowires are particularly relevant to antimicrobial surfaces because they mimic vanadium haloperoxidases and catalyze the oxidation of halides to antimicrobial hypohalous species, thereby suppressing biofilm formation [13]. This haloperoxidase-like route is important because it differs from conventional Fenton-like hydroxyl radical generation and may offer alternative antimicrobial pathways for wet processing environments.

Antimicrobial nanozymes generally operate through peroxidase-, oxidase-, catalase-, superoxide dismutase-, haloperoxidase-, or multi-enzyme-like activities. Peroxidase-like nanozymes catalyze H_2_O_2_ decomposition to generate hydroxyl radicals or related oxidative species, which can damage bacterial membranes, proteins, lipids, and nucleic acids [33]. Oxidase-like nanozymes directly activate dissolved oxygen, which is attractive for applications where external H_2_O_2_ addition is undesirable [34]. Catalase- and superoxide dismutase-like activities, by contrast, can either protect host tissues or reduce antimicrobial oxidative pressure, depending on the intended application [35]. This duality is especially clear for CeO_2_ nanozymes, which can show both pro-oxidant and antioxidant behavior depending on particle size, surface chemistry, Ce^3+^/Ce^4+^ ratio, oxygen vacancy concentration, and pH [36,37]. Therefore, the antimicrobial value of nanozyme cannot be judged only by the presence of enzyme-like activity, depending on whether the catalytic pathway produces the desired biological outcome under food-relevant conditions.

### 3.2. Structure–Activity Determinants Governing Antimicrobial Catalysis

The antimicrobial performance of catalytic nanozymes is governed by structure–activity relationships linking nanoscale architecture to catalytic efficiency, ROS generation, bacterial interaction, and biofilm disruption. Composition is the most direct determinant because the redox potential, coordination environment, and electron-transfer capacity of active sites define the catalytic pathway [38]. However, composition alone is insufficient to explain antimicrobial activity. Particle size, morphology, exposed facets, defect density, surface charge, ligand chemistry, porosity, and heterointerfaces can substantially alter substrate adsorption, H_2_O_2_ activation, oxygen reduction, and bacterial contact [39,40].

Defect engineering is one of the most important strategies for enhancing antimicrobial nanozyme activity. Defects can introduce unsaturated coordination sites, improve electron transfer, increase surface adsorption, and regulate ROS generation. A defect-rich Fe_3_O_4_@MoS_2_–Ag nanozyme showed enhanced peroxidase-like activity and broad-spectrum antibacterial performance through the combined effects of ROS production, Ag^+^ release, photothermal activity, and bacteria-binding ability [41]. In line with prior developments in metal-free defect engineering, the introduction of abundant nitrogen vacancies in carbon nitride quantum dots has been shown to markedly enhance peroxidase-like catalytic activity, resulting in potent, broad-spectrum microbicidal effects [42]. Moreover, the catalytic versatility of these nanozymes can be finely tuned through structural hybridization. As demonstrated by incorporating attapulgite into MoS_2_-based nanostructures, establishing a supportive matrix not only enhances antibacterial activity but also improves glutathione detection, underscoring the critical role of interfacial modulation in catalytic optimization [43].

Single-atom engineering has recently provided a more precise route to define catalytically active sites. In single-atom nanozymes, isolated metal centers coordinated with nitrogen, carbon, oxygen, or sulfur atoms can maximize atom utilization and create enzyme-like coordination environments. Copper single-atom catalysts have been reported to combine photothermal conversion with enhanced nanozyme activity for antibacterial therapy [44]. Spherical mesoporous Fe–N–C single-atom nanozymes improved Fenton-like antibacterial catalysis under photothermal activation, while Mn single-atom catalysts exhibited catalytic–photothermal synergistic antibacterial effects [18,45]. Qu et al. (2024) explored that the Cu/Mn dual single-atom nanozyme exhibited superior antibacterial activity against multidrug-resistant bacteria through synergistic peroxidase-like catalysis and mild photothermal therapy [46]. These findings provide useful mechanistic guidance for the design of antimicrobial nanozymes intended for food applications. A similar selectivity can be achieved under complex food-processing conditions that remain to be established.

**Figure 1:**
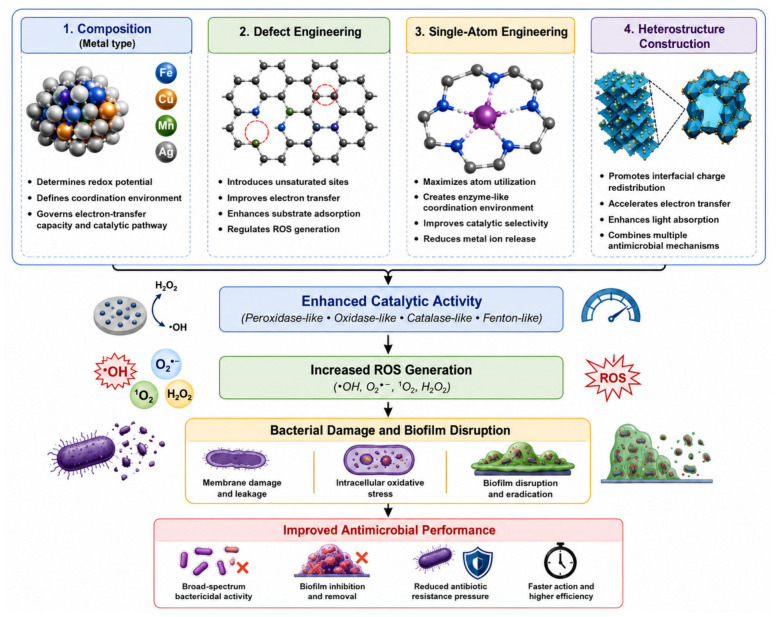
Engineering strategies for catalytic nanozymes and their mechanisms of ROS-mediated antimicrobial activity. The schematic illustrates the antimicrobial mechanisms under simplified experimental conditions. In practical food systems, intrinsic food components, including proteins, lipids, and phenolic compounds, may scavenge reactive oxygen species (ROS), adsorb onto nanozyme surfaces, or block catalytic active sites, thereby reducing antimicrobial efficacy.

Heterostructure construction is another powerful design strategy. By coupling two or more components, heterostructures can create interfacial charge redistribution, accelerate electron transfer, improve light absorption, or combine multiple antimicrobial mechanisms [47]. MXene-based single-atom catalytic platforms and Ag-containing heterostructure nanozymes have demonstrated amplified peroxidase-like activity, photothermal enhancement, and controlled metal release for synergistic antibacterial action [19,48]. MOF-derived or MOF-based nanozymes add a further layer of tunability because metal nodes, organic linkers, pore architecture, and dimensionality can be rationally regulated. Dimensionality reduction in bimetallic MOF nanozymes, for instance, was shown to boost peroxidase-like activity and improve antibacterial performance against multidrug-resistant bacteria [49]. These studies indicate that antimicrobial nanozyme engineering is moving from material discovery toward active-site design, where catalytic output can be adjusted through structural control. The major structure–activity relationships governing antimicrobial nanozyme performance are summarized in Figure 1.

### 3.3. Relevance of Nanozyme Design to Food Antimicrobial Applications

For antimicrobial food systems, catalytic nanozyme design must be evaluated beyond standard planktonic bacterial assays. Foodborne bacteria often persist on stainless steel, plastic, rubber, conveyor belts, cutting surfaces, fresh produce, meat, seafood, and packaging interfaces [50]. In these environments, antimicrobial performance is strongly affected by surface roughness, organic load, water activity, pH, salt concentration, proteins, lipids, carbohydrates, and phenolic compounds. These matrix components may consume ROS, adsorb nanozyme surfaces, block catalytic sites, alter nanoparticle dispersion, or reduce bacterial contact [51]. Therefore, nanozyme activity measured using model substrates such as TMB or ABTS cannot be directly translated into food antimicrobial efficacy without validation in realistic systems.

This limitation is increasingly recognized in food nanozyme research because antimicrobial performance measured in simple buffer systems often overestimates efficacy under practical food conditions. Antimicrobial applications face greater challenges than analytical detection because food components actively interfere with catalytic reactions. Proteins can adsorb onto nanozyme surfaces to form a biomolecular corona that blocks catalytic active sites, while lipids and emulsified fat droplets hinder nanozyme–bacteria interactions [52]. In addition, endogenous phenolic compounds, vitamins, and other natural antioxidants present in fruit juices and plant-derived foods readily scavenge reactive oxygen species (ROS), thereby reducing oxidative damage to bacterial cells. These matrix-dependent effects may substantially diminish ROS availability, decrease catalytic efficiency, and ultimately reduce antibacterial performance compared with buffer-based assays. Future antimicrobial nanozyme studies should routinely validate efficacy in representative food matrices to establish realistic performance under industrial conditions.

Current food-related nanozyme research has been most active in pathogen detection, where catalytic signal amplification can improve sensitivity and shorten assay time. Pt nanoparticle-modified Zr-MOF nanozymes have been integrated into microfluidic biosensing platforms for *E. coli* O157 detection in water, milk, and cabbage samples [20]. Bifunctional nanozyme-mediated catalytic amplification combined with SERS has enabled ultrasensitive pathogen detection [53]. Prussian blue nanozyme labels have also been used for simultaneous immunochromatographic detection of *Salmonella typhimurium* and *Listeria monocytogenes* in milk [22]. A Pt–Au bimetallic nanoparticle-based immunochromatographic assay enabled rapid, visual, and quantitative detection of *E. coli* O157:H7 through peroxidase-mediated signal amplification [54]. Meanwhile, Wang et al. (2020) synthesized a label-free, multi-readout lateral flow immunoassay employing mannose-modified Prussian blue nanozymes, enabling rapid detection of *E. coli* O157:H7 through a nanozyme–bacteria–antibody sandwich format [55]. The assay combined intrinsic strip signals with catalytic colorimetric amplification, achieving a detection range of 10^2^–10^8^ CFU/mL and a detection limit of 10^2^ CFU/mL in real samples.

More integrated platforms have begun combining detection with on-demand disinfection, such as multifunctional nanozyme systems for *L. monocytogenes* monitoring and elimination [56]. These examples demonstrate that nanozymes can contribute not only to microbial killing but also to rapid decision-making in food safety management.

Nanozyme-based preservation and coating systems are also emerging. Chitosan composite Ce-MOF nanozyme coatings have been developed for fruit preservation, showing antibacterial activity against *E. coli* and *S. aureus* while improving the storage performance of strawberries and bananas [24]. H_2_O_2_-activatable peptide nanozyme–alginate hydrogel coatings have further shown how catalytic antimicrobial function can be integrated with moisture retention for postharvest preservation [25]. Magnetically retained PDA/Fe_3_O_4_ nanozymes achieved efficient antibacterial chemodynamic therapy by depleting glutathione, generating hydrogen peroxide and hydroxyl radicals, and enhancing peroxidase-like activity through photothermal heating [57]. The nanozymes effectively eliminated *Staphylococcus aureus* and *Escherichia coli*, promoted localized wound disinfection, minimized healthy tissue damage, and demonstrated excellent biosafety. These studies represent important progress toward practical food applications by evaluating nanozymes under more representative food conditions. Additional validation across diverse food matrices is still required before industrial implementation. However, they also reveal major translational requirements. Antimicrobial nanozymes for food systems must maintain activity under variable pH, temperature, humidity, and nutrient conditions; avoid undesirable sensory changes; minimize nanoparticle migration; and remain compatible with existing processing equipment and packaging lines. As summarized in Table 1, current nanozyme research has expanded from pathogen detection toward multifunctional preservation and antimicrobial applications, while highlighting key translational challenges.

Safe-by-design considerations are therefore inseparable from structure–activity optimization. Nanomaterials incorporated into food-contact systems may migrate depending on particle size, matrix composition, storage temperature, contact time, and food simulant chemistry. Recent research on safe-and-sustainable-by-design food packaging further emphasizes that antimicrobial function must be balanced with exposure control, environmental behavior, and end-of-life considerations [29]. Thus, the most promising antimicrobial nanozymes for food applications are not necessarily those with the highest catalytic activity in vitro, but those that achieve a balanced profile of catalytic efficacy, interfacial stability, low migration risk, controlled ROS generation, scalable fabrication, and regulatory plausibility. This design logic provides the foundation for the following sections, which examine antimicrobial mechanisms, application-specific performance, and translation barriers in greater depth.

## 4. Catalytic Antimicrobial Mechanisms of Nanozymes in Food-Related Systems

### 4.1. ROS-Mediated Catalytic Oxidation and Microenvironment-Activated Killing

The most widely reported antimicrobial mechanism of catalytic nanozymes is the localized generation or regulation of reactive oxygen species (ROS). Peroxidase-like nanozymes catalyze the conversion of hydrogen peroxide into highly oxidative species, particularly hydroxyl radicals, which can damage bacterial membranes, proteins, nucleic acids, and metabolic enzymes [58]. This catalytic process is especially relevant to food systems because low concentrations of H_2_O_2_, organic acids, or endogenous oxidative species may be present during sanitation, postharvest preservation, or active packaging. Compared with direct addition of oxidants, nanozyme-mediated ROS generation can provide localized and sustained oxidative pressure at the pathogen–material interface, potentially reducing the amount of free chemical sanitizer required [59].

**Figure 2:**
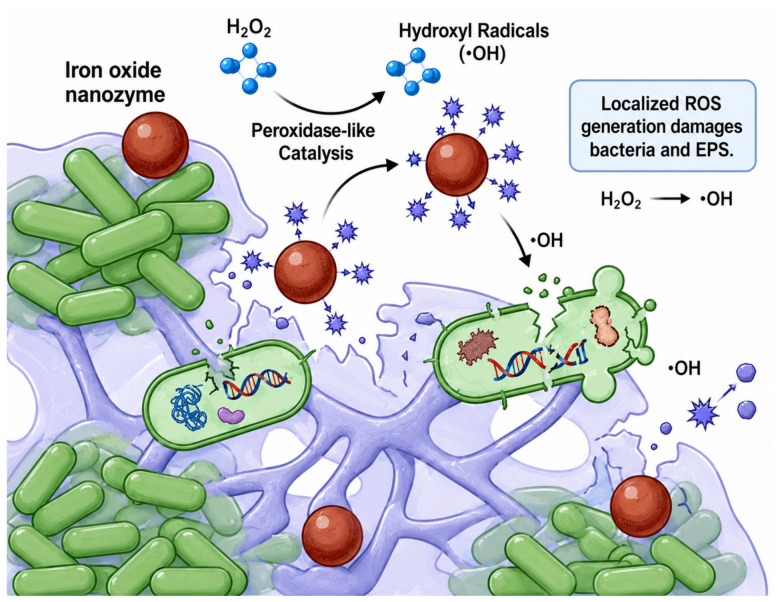
Schematic illustration of the peroxidase-like catalytic mechanism of iron oxide nanozymes for localized ROS generation, bacterial damage, and biofilm disruption.

Beyond intrinsic oxidase-like activity, many nanozymes exhibit peroxidase-like catalytic behavior by activating endogenous or externally supplied H_2_O_2_ to generate highly reactive hydroxyl radicals (•OH). The localized production of ROS within bacterial biofilms induces oxidative damage to extracellular polymeric substances (EPS), disrupts cell membranes, and damages intracellular biomolecules, ultimately leading to biofilm eradication and bacterial death (Figure 2).

Iron oxide nanozymes are among the most representative platforms for ROS-mediated antimicrobial activity. Ferumoxytol nanoparticles were shown to bind within biofilm structures and catalyze H_2_O_2_ conversion into free radicals, causing bacterial membrane damage and EPS matrix degradation [14,60]. In a human-derived biofilm model, ferumoxytol combined with low-level H_2_O_2_ reduced biofilm accumulation and prevented acid-mediated surface damage, demonstrating that catalytic ROS generation can be effective in complex biological matrices [45]. Although these studies were conducted in oral biofilm systems, they provide valuable mechanistic insights into ROS-mediated biofilm disruption. However, the antibacterial performance of nanozymes should be validated under realistic food-contact conditions, where food matrices and environmental factors may substantially influence catalytic activity.

A major concern for ROS-based antimicrobial systems is selectivity, because ROS can also damage mammalian cells or food components [61]. Surface-bound ROS-generating nanozymes provide an important solution by producing reactive intermediates with limited diffusion distances. Gao et al. (2021) demonstrated that nanozymes generating surface-confined ROS selectively killed bacteria over mammalian cells, partly because bacteria lack endocytosis-mediated internalization pathways that can reduce contact with surface-bound oxidants [15]. This finding is important for antimicrobial food systems because it suggests that spatial control of ROS generation, rather than simply maximizing ROS concentration, may improve safety and functional selectivity. Therefore, future food-directed nanozymes should be evaluated not only by total ROS yield but also by ROS type, lifetime, diffusion distance, localization, and reactivity with food matrix components.

Beyond hydroxyl radicals, haloperoxidase-like nanozymes represent another antimicrobial catalytic route. V_2_O_5_ nanowires mimic vanadium haloperoxidases and catalyze the oxidation of bromide ions in the presence of H_2_O_2_ to generate hypobromous acid, thereby preventing biofilm formation [13]. CeO_2_−x nanorods embedded in nanofibrous mats also showed haloperoxidase-like activity and converted bromide and H_2_O_2_ into antimicrobial hypobromous acid [62]. Henych et al. (2023) determined that Palmitate-induced lipotoxicity impaired intestinal organoid growth, reducing stem cell proliferation and altering epithelial differentiation [63]. These systems may have potential for food-processing environments where wet surfaces, salts, and halide ions are present. Nevertheless, their practical applicability requires validation under realistic food-contact conditions.

### 4.2. Cell Envelope Damage, Ion Release, and Intracellular Metabolic Disruption

**Figure 3:**
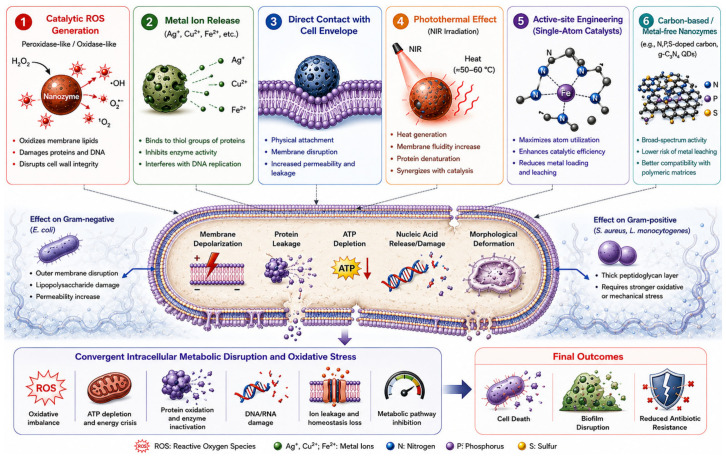
Multifactorial antibacterial mechanisms of engineered nanozymes beyond reactive oxygen species generation. The schematic represents the principal antibacterial pathways under simplified experimental conditions. In real food matrices, proteins, lipids, phenolic compounds, and other organic constituents may attenuate ROS activity, interfere with nanozyme–bacteria interactions, or partially block catalytic active sites, resulting in reduced antibacterial performance.

Nanozyme-mediated antibacterial activity is not limited to ROS generation. Many nanozymes damage bacteria through combined catalytic, physicochemical, and interfacial mechanisms (Figure 3).

The bacterial cell envelope is often the first target because it directly contacts the nanozyme surface. ROS generated at or near the nanozyme–cell interface can oxidize membrane lipids, disrupt cell-wall integrity, increase permeability, and cause leakage of intracellular components [64]. These effects are frequently observed through membrane depolarization, protein leakage, ATP depletion, nucleic acid release, and morphological deformation under electron microscopy. Gram-negative bacteria such as *E. coli* may be affected through outer membrane disruption and lipopolysaccharide damage, whereas Gram-positive bacteria such as *S. aureus* and *L. monocytogenes* may require stronger oxidative or mechanical stress because of their thicker peptidoglycan layer [65].

Hybrid nanozymes often enhance antibacterial activity by combining catalytic ROS generation with ion release, photothermal effects, or direct membrane contact. A defect-rich Fe_3_O_4_@MoS_2_–Ag nanozyme exhibited peroxidase-like activity, Ag^+^ release, photothermal conversion, and bacterial binding, resulting in synergistic antibacterial activity against *E. coli* [41]. The study showed that the antibacterial process was not governed by a single pathway but by the coordinated effects of ROS, silver ion toxicity, and near-infrared-induced thermal stress. Similarly, heterostructure nanozymes with controlled silver release and hyperthermia-amplified enzyme-like activity have been designed for synergistic bacterial killing [48]. Such multimodal designs are attractive because they reduce reliance on a single antimicrobial mechanism, which may be useful against stress-adapted bacteria and biofilms.

Single-atom nanozymes further illustrate how active-site engineering can alter antimicrobial mechanisms. Copper single-atom catalysts have been reported to integrate photothermal performance with enhanced nanozyme activity for bacterial infection therapy [44]. Fe–N–C single-atom nanozymes showed catalytic–photothermal synergistic antibacterial effects, while Mn single-atom catalysts demonstrated combined catalytic and photothermal activity against infected tissues [18,45]. Although these examples are largely biomedical, they provide useful mechanistic principles for food systems: atomically dispersed sites can maximize catalytic efficiency, reduce metal loading, and enable better control over redox reactions. For food applications, these advantages could support lower-dose antimicrobial materials, provided that migration, toxicity, and food-contact safety are carefully evaluated.

Carbon-based and metal-free nanozymes provide another promising route to reduce concerns associated with metal leaching. Carbon nitride quantum dots with abundant nitrogen vacancies showed enhanced peroxidase-like activity and broad-spectrum antibacterial performance [42]. N, P, and S co-doped carbon nanozymes have also been developed to improve enzyme-mimicking behavior and antibacterial function [66]. These materials may be particularly valuable for food packaging and coatings because their composition can be more compatible with polymeric or biopolymeric matrices. However, metal-free does not automatically mean risk-free; their persistence, surface reactivity, oxidative behavior, and potential effects on gut or environmental microbiota still require systematic evaluation.

### 4.3. EPS Degradation, Biofilm Eradication, and Food-Contact Surface

Biofilms are among the most difficult microbial targets in food systems because bacterial cells are protected by EPS composed of polysaccharides, proteins, extracellular DNA, lipids, and trapped nutrients. EPS reduces antimicrobial penetration, buffers oxidative stress, supports microbial adhesion, and promotes resistance to cleaning procedures. Nanozymes are promising antibiofilm agents because they can catalytically generate reactive species within the biofilm microenvironment and degrade both bacterial cells and matrix components [62]. Unlike conventional disinfectants that may be rapidly neutralized by organic matter, nanozymes can potentially sustain local catalytic activity as long as substrates such as H_2_O_2_, dissolved oxygen, or halides are available.

Ferumoxytol provides a representative example of matrix-targeted antibiofilm catalysis. The nanoparticles localized within biofilm structures and generated radicals from H_2_O_2_, resulting in bacterial killing and EPS degradation [14]. This dual action is important because biofilm control requires not only killing cells but also dismantling the protective matrix to prevent regrowth and reattachment. V_2_O_5_ and CeO_2−x_ haloperoxidase-like nanozymes similarly suppressed biofilm formation by catalytically producing antimicrobial hypohalous species [13]. In another example, dimensionality-reduced bimetallic MOF nanozymes exhibited enhanced peroxidase-like activity and improved eradication of multidrug-resistant bacteria, supporting the idea that structural engineering can strengthen antibiofilm activity [49].

In food-related applications, nanozyme-based strategies are beginning to move from model antibacterial assays toward realistic preservation and detection systems. Chitosan composite Ce-MOF nanozyme spray coatings showed oxidase- and apyrase-like activities, inhibited *E. coli* and *S. aureus*, and improved storage quality of strawberries and bananas [24]. H_2_O_2_-activatable peptide nanozyme–alginate dual-network hydrogel coatings were designed to combine antibacterial activity with moisture retention during postharvest preservation [25]. Ding et al. (2022) performed a dual-mode agarose hydrogel containing silver-doped Prussian blue nanoparticles, which enabled colorimetric and photothermal detection of trimethylamine for monitoring shrimp and fish freshness [67]. Both signals showed linear responses between 0.21 and 0.54 ppm TMA. The photothermal readout remained reliable even when colorimetric detection was hindered by food coloration, improving practical on-site freshness assessment. Rather than functioning only as suspended antimicrobial particles, catalytic nanozymes can integrate into food preservation systems, biosensors, hydrogels, and coatings.

For industrial food-contact surfaces, the most important design goal is controlled catalytic activity at the interface. Stainless steel, rubber, plastic, and conveyor surfaces are repeatedly exposed to water, organic residues, sanitizers, and mechanical abrasion. Therefore, immobilized nanozyme coatings may be more practical than freely dispersed nanoparticles because immobilization can reduce migration, improve recoverability, and localize catalytic activity [68]. Haloperoxidase-like nanozymes embedded in polymer mats provide an instructive model for antifouling surfaces because the catalytic particles remain fixed while producing antimicrobial species at the material interface [69]. Similar logic could be applied to food-processing equipment, but future work must validate durability after repeated washing, sanitizer exposure, mechanical wear, and contact with real foods.

Inclusively, nanozyme antimicrobial mechanisms are best understood as multi-layered processes involving catalytic ROS generation, interfacial bacterial contact, membrane disruption, intracellular oxidation, EPS degradation, and biofilm detachment. The most effective systems are unlikely to be those that simply maximize ROS production; rather, they should generate the right reactive species at the right location, at a controlled rate, and within a safe exposure window. This mechanistic understanding is essential for translating catalytic nanozymes from laboratory antibacterial assays into antimicrobial food systems that are effective, safe, scalable, and compatible with industrial processing.

## 5. Application-Oriented Performance of Catalytic Nanozymes in Antimicrobial Food Systems

### 5.1. Nanozyme-Enabled Detection and Monitoring of Foodborne Pathogens

Rapid and sensitive detection of foodborne pathogens is one of the most mature food-related applications of catalytic nanozymes [70]. Conventional culture-based assays remain reliable but are often time-consuming, while polymerase chain reaction and immunoassays may require expensive instrumentation, trained personnel, or enzyme labels with limited stability. Nanozymes address several of these limitations by functioning as robust catalytic signal amplifiers in colorimetric, electrochemical, photothermal, chemiluminescent, photoelectrochemical, and surface-enhanced Raman scattering (SERS) platforms. Their enzyme-mimicking activity can replace natural horseradish peroxidase or alkaline phosphatase, thereby improving operational stability and reducing assay cost under field-relevant food testing conditions [20,54].

Prussian blue nanoparticles are among the most widely used nanozyme labels for foodborne pathogen detection because of their strong peroxidase-like activity, intrinsic blue color, and compatibility with lateral flow formats. Wang et al. (2020) developed a mannose-modified Prussian blue nanozyme for multi-readout and label-free lateral flow detection of *Escherichia coli* O157, in which the nanozyme acted simultaneously as a recognition agent and catalytic signal indicator [54]. This design is especially relevant for food safety because mannose modification enabled direct affinity toward bacterial flagella, reducing dependence on antibody labeling. Similarly, Prussian blue nanozyme-enhanced immunochromatographic assays have been developed for simultaneous detection of *Salmonella typhimurium* and *Listeria monocytogenes* in milk, demonstrating the potential of catalytic labels for multiplex pathogen control in complex food matrices [22]. Au@Pt nanozymes have also been used in immunochromatographic assays for *Salmonella typhimurium*, where peroxidase-like amplification improved visual sensitivity compared with traditional gold nanoparticle labels [22]. Hong et al. (2021) demonstrated a gold nanoparticle-assisted magnetic relaxation sensor enabled ultrasensitive detection of aflatoxin B1 through triple cascade signal amplification involving nanoparticle-enriched initiator DNA, hybridization chain reaction [71]. A report of Song et al. (2022) stated that a single-atom Ce-N-C nanozyme with enhanced peroxidase-like activity enabled rapid, portable pesticide detection through cascade catalysis with acetylcholinesterase [72]. Combined with bioactive paper and a 3D-printed platform, it detected four organophosphate and carbamate pesticides within 30 min, achieving detection limits of 55.83–81.81 ng/mL and recoveries of 84.09–104.68%.

Microfluidic nanozyme biosensors represent another important direction because they integrate sample pretreatment, enrichment, immunoreaction, washing, and catalytic readout within compact devices. Xing et al. (2023) developed a Pt nanoparticle-modified Zr-MOF nanozyme-mediated microfluidic biosensor for *E. coli* O157 detection in water, milk, and cabbage samples, achieving rapid detection within one hour and good agreement with standard culture methods [20]. Cheng et al. (2025) later reported a nanozyme-catalyzed colorimetric microfluidic immunosensor using porous Au@Pt nanopompoms for filtration enrichment and ultrasensitive detection of *Salmonella typhimurium* in food samples [73]. These examples highlight the practical value of combining nanozyme catalytic amplification with portable microfluidic formats, particularly for on-site screening in food processing, cold-chain logistics, and quality-control laboratories. Representative examples of nanozyme-enabled sensing platforms for foodborne pathogens and food contaminants are summarized in Table 2, highlighting the diversity of catalytic materials, sensing formats, and practical food applications.

Beyond colorimetric detection, catalytic nanozymes are increasingly integrated with SERS and dual-mode sensing to improve accuracy. Li et al. (2025) combined bifunctional nanozyme-mediated catalytic signal amplification with label-free SERS immunoassays for ultrasensitive pathogen detection in milk samples [55]. Dual-mode sensors have also been developed for *L. monocytogenes*, including photoelectrochemical and colorimetric platforms based on TiO_2_–Fe–N–C nanozymes. More advanced detect-and-eliminate systems have been reported, such as multifunctional nanozyme platforms capable of ultra-accurate detection and on-demand disinfection of *L. monocytogenes* [74]. These integrated strategies are particularly attractive for antimicrobial food systems because they move beyond passive detection toward responsive control, where pathogen identification can be coupled with localized antimicrobial action.

### 5.2. Food-Contact Surface Decontamination and Biofilm Control

Catalytic nanozymes offer a promising approach for surface decontamination because they can generate antimicrobial reactive species directly at the material–microbe interface. Compared with freely diffusing disinfectants, immobilized or recoverable nanozymes may provide localized catalytic activity, improved reusability, and lower risk of uncontrolled release.

Haloperoxidase-like nanozymes provide a useful model for catalytic antibiofilm surfaces. Vanadium pentoxide nanowires were shown to mimic vanadium haloperoxidases and prevent biofilm formation by catalyzing the production of antimicrobial hypohalous species [33]. Hu et al. further incorporated haloperoxidase-like nanozymes into nanofibrous mats, creating antifouling surfaces capable of combating biofilm formation through localized catalytic activity [62]. Although these systems were not designed exclusively for food-processing surfaces, their design principles are highly relevant because wet food-processing environments often contain salts, low-level oxidants, and organic residues that may support catalytic antimicrobial reactions.

Recoverable magnetic nanozymes are also attractive for surface sanitation because they can be applied, activated, and removed after treatment. Fe_3_O_4_-based nanozymes have been widely studied because their peroxidase-like activity can convert H_2_O_2_ into hydroxyl radicals, while their magnetic properties facilitate separation and reuse [75]. Hybrid systems can further enhance antimicrobial performance. For example, Fe_3_O_4_@MoS_2_–Ag nanozymes exhibited recoverable peroxidase-like activity and enhanced antibacterial effects through combined ROS generation, silver ion release, photothermal conversion, and bacterial binding [41]. Dimensionality-reduced bimetallic MOF nanozymes have also been shown to enhance peroxidase-like activity and improve eradication of multidrug-resistant bacteria [76]. These findings suggest that food-contact surface applications should prioritize immobilized, magnetically recoverable, or coating-integrated nanozymes rather than freely dispersed nanoparticles.

Biofilm control requires both bacterial killing and matrix disruption. Ferumoxytol nanozymes have demonstrated the ability to localize within biofilms and catalyze H_2_O_2_-mediated radical generation, resulting in simultaneous bacterial inactivation and extracellular matrix degradation [60,77]. Surface-bound ROS-generating nanozymes further show that localized oxidative stress can selectively kill bacteria while limiting off-target damage to mammalian cells [15]. This principle is relevant for food-contact surfaces because ROS localization may reduce the amount of chemical oxidant required and improve compatibility with equipment materials. Ferumoxytol nanozymes combined with hydrogen peroxide effectively eradicated chronic endodontic biofilms, achieving antibacterial efficacy comparable to sodium hypochlorite without detectable cytotoxicity or adverse effects [60]. The nanozymes preferentially targeted pathogenic bacteria while promoting stem cell proliferation, pluripotency, and osteogenic differentiation. Clinical treatment achieved approximately 99.9% bacterial reduction, highlighting their dual antimicrobial and regenerative therapeutic potential [78,79]. However, direct translation to food-processing environments requires validation against foodborne biofilms formed by *Listeria monocytogenes*, *Salmonella enterica*, *E. coli* O157, *Pseudomonas* spp., and mixed microbial communities under realistic organic-load conditions.

### 5.3. Active Packaging, Edible Coatings, and Food Preservation

The area of active packaging and edible coatings represents one of the most promising application fields for antimicrobial nanozymes because these systems can integrate catalytic activity with barrier function, moisture regulation, gas exchange, and postharvest quality preservation. In contrast to conventional antimicrobial packaging that relies on passive release of preservatives, nanozyme-based materials can be designed to generate antimicrobial activity in response to environmental triggers such as H_2_O_2_, oxygen, pH change, humidity, or microbial contamination. This responsive behavior is particularly valuable for fresh produce, minimally processed foods, and refrigerated products, where microbial growth and quality deterioration occur gradually during storage.

Nanocellulose-based polymeric nanozymes have been developed as bioinspired spray coatings for fruit preservation, demonstrating how enzyme-like catalytic activity can be incorporated into biopolymer-based preservation systems [80]. Chitosan composite Ce-MOF nanozyme coatings have also been reported for fruit preservation, where oxidase-like and apyrase-like activities contributed to antibacterial effects and delayed quality loss in strawberries and bananas [81]. More recently, antimicrobial packaging films based on MOF nanozymes engineered via biomineralization and bimetallic hybridization were developed for fruit preservation, showing that catalytic nanozyme structures can be embedded into packaging films rather than used only as surface sprays [82]. Jia et al. (2021) determined a mussel-inspired tannic acid-chelated silver nanozyme-enabled self-setting adhesive hydrogels through peroxidase-like catalysis without external initiators [83]. The hydrogel exhibited long-lasting adhesion, enhanced mechanical strength, electrical conductivity, and potent antibacterial activity via reactive oxygen species generation and silver ions. These examples indicate that nanozyme-enabled food packaging is beginning to move from proof-of-concept antimicrobial assays toward integrated preservation materials.

Hydrogel coatings provide another useful platform because they can retain moisture while delivering catalytic antimicrobial activity. Li et al. (2026) developed H_2_O_2_-activatable peptide nanozyme–alginate dual-network hydrogel coatings for strawberry preservation, aiming to reconcile antibacterial activity with moisture retention [25]. This design is significant because postharvest preservation requires more than microbial inhibition; it must also maintain firmness, reduce weight loss, limit oxidative deterioration, and preserve sensory quality. Peptide nanozymes may offer additional advantages over inorganic particles because their composition can be more biodegradable and potentially more acceptable for food-contact use, although systematic safety assessment remains necessary [84]. Examples of nanozyme-enabled packaging and coating systems developed for food preservation are summarized in Table 3, illustrating the diversity of material platforms, preservation strategies, and functional advantages reported to date.

Despite these advances, several translational issues remain unresolved. First, many nanozyme packaging and coating studies use model pathogens or simplified storage conditions, while real foods contain complex microbial communities and variable physicochemical conditions. Second, catalytic activity may be reduced by proteins, phenolics, lipids, sugars, organic acids, and salts that adsorb onto nanozyme surfaces or scavenge ROS. Third, food packaging applications require careful control of nanoparticle migration, ion release, sensory effects, and environmental fate [80,85]. Therefore, the success of antimicrobial nanozymes in food preservation will depend on balanced design: sufficient catalytic activity to suppress microbial growth, strong immobilization to minimize migration, compatibility with edible or food-contact matrices, and preservation of food quality throughout storage. Future research should move toward standardized testing in real food matrices, repeated-use surface models, mixed-species biofilms, migration assays, sensory evaluation, and pilot-scale packaging or coating trials.

## 6. Safe-by-Design Principles and Industrial Translation of Catalytic Nanozymes

### 6.1. Designing Antimicrobial Nanozymes for Controlled Exposure and Food-Contact Safety

The industrial translation of catalytic nanozymes in antimicrobial food systems requires a shift from activity-centered development to exposure-aware material design. In early-stage studies, nanozymes are often optimized for catalytic efficiency, pathogen inhibition, or biofilm reduction. However, food applications introduce a distinct exposure scenario because consumers may be repeatedly exposed to materials through direct food contact, packaging interaction, edible coatings, or residues from processing surfaces. Therefore, safe-by-design development should begin by defining the intended use category: removable sanitation aid, immobilized food-contact coating, active packaging component, edible coating, or sensing element. Each category requires a different exposure-control strategy.

For immobilized food-contact materials, the key design objective is to retain catalytic function while minimizing particle release. Migration studies of nano-enabled food-contact materials have shown that release behavior can depend on the polymer matrix, particle chemistry, storage time, food-simulant composition, temperature, and repeated contact cycles [86]. For example, silver migration from antimicrobial food containers and polymeric food-contact materials has been measured under different simulant conditions, showing that detectable migration may occur as ionic silver, particulate silver, or both, depending on product composition and analytical method [87,88]. These findings are relevant to nanozymes because catalytic antimicrobial performance is often linked to active metals, redox cycling, and surface reactivity. Materials that perform well microbiologically may still be unsuitable for food contact if they exhibit uncontrolled migration, metal leaching, or instability during repeated washing and storage.

A safe-by-design approach should therefore integrate multiple material-level controls: strong matrix immobilization, surface passivation when appropriate, reduced metal loading, selection of low-toxicity components, controlled dissolution, and use of biodegradable or food-compatible carriers [89]. For nanozyme coatings, covalent anchoring, polymer entrapment, layer-by-layer assembly, hydrogel confinement, and magnetic recoverability may reduce uncontrolled particle transfer. For active packaging, nanozymes should be embedded within matrices that allow controlled catalytic interaction at the interface without excessive release into foods. For edible coatings, safety requirements are even stricter because ingestion is expected; therefore, material composition, degradation products, gastrointestinal transformation, and microbiome interactions should be evaluated.

Importantly, safety assessment should not be limited to acute cytotoxicity. Catalytic nanozymes may alter oxidative balance, release ions, interact with proteins, adsorb nutrients or bioactive compounds, or transform during digestion and storage. Therefore, future studies should include physicochemical characterization before and after food contact, ion-release profiling, migration testing in appropriate simulants and real foods, gastrointestinal stability, intestinal barrier models, genotoxicity screening, and environmental fate evaluation. For catalytic materials, residual activity after migration or degradation should also be considered, because a transformed particle may retain or lose enzyme-like behavior. Thus, the central safe-by-design question is not only whether a nanozyme is toxic, but whether its exposure, transformation, catalytic persistence, and degradation products remain acceptable across the intended life cycle.

### 6.2. Regulatory Readiness, Manufacturing Scalability, and Standardized Evaluation

Food-contact safety evaluation of nanozymes requires systematic assessment of nanoparticle migration under conditions representative of intended use. Regulatory migration testing commonly employs four categories of food simulants to represent the major physicochemical characteristics of foods: aqueous simulants for water-based foods, acidic simulants for products containing organic acids, alcoholic simulants for beverages and foods containing ethanol, and oily simulants for fatty foods. These standardized simulants enable conservative and reproducible estimation of nanoparticle and metal-ion migration across a broad range of food types while facilitating comparison among different materials and studies. Regulatory readiness is a major barrier for antimicrobial nanozymes intended for food systems. In the United States, FDA guidance emphasizes case-by-case consideration of products involving nanotechnology, particularly when changes in particle size, surface area, morphology, or other nanoscale properties may alter safety or regulatory status [90]. In Europe, EFSA guidance provides a structured pathway for risk assessment of nanomaterials in the food and feed chain, including applications related to food contact materials, additives, novel foods, and pesticides [91]. These frameworks make clear that nanoscale materials cannot be evaluated only by bulk composition; their particle identity, size distribution, aggregation state, dissolution behavior, surface chemistry, and exposure profile must be characterized.

For catalytic nanozymes, regulatory translation is especially complex because their function depends on surface reactivity. Unlike inert fillers, nanozymes are designed to participate in redox reactions, generate or regulate reactive species, and interact with microorganisms or food components. Therefore, standardized evaluation should include both conventional nanomaterial characterization and catalytic performance metrics [92]. At a minimum, studies should report primary particle size, hydrodynamic size, zeta potential, morphology, crystallinity, specific surface area, oxidation state, surface functional groups, active-site composition, catalytic kinetics, metal leaching, aggregation behavior, and stability under food-relevant pH, salt, temperature, and organic-load conditions [93]. The OECD nanomaterial guidelines for particle-size distribution and dispersion stability provide useful reference points for harmonizing characterization, but food-specific catalytic testing remains underdeveloped [94].

Manufacturing scalability is another under-discussed issue. Many high-performing nanozymes are synthesized using laboratory-scale hydrothermal reactions, noble-metal deposition, multi-step ligand functionalization, or complex heterostructure assembly. These routes may be difficult to scale economically or consistently. Industrial translation requires reproducible batch-to-batch catalytic activity, low-cost precursors, solvent and energy efficiency, minimal hazardous reagents, compatibility with coating or film-forming processes, and quality-control assays suitable for production environments. Scalable technologies such as aqueous synthesis, continuous flow synthesis, spray coating, melt compounding, extrusion, electrospinning, and roll-to-roll coating should therefore receive greater attention. Despite the rapid expansion of nanozyme research, nearly all antimicrobial nanozyme platforms summarized in Table 1, Table 2 and Table 3 remain at the laboratory or proof-of-concept stage, with limited demonstration at pilot or industrial scales. Their translational readiness varies considerably depending on material complexity, synthesis reproducibility, manufacturing cost, and regulatory feasibility. Highly engineered systems, such as single-atom catalysts, defect-rich heterostructures, and multifunctional hybrid nanozymes, often require sophisticated synthesis procedures and stringent quality control, which currently limit large-scale production [95]. In contrast, several nanozyme platforms appear more promising for industrial translation. Nevertheless, successful industrial implementation will require standardized production protocols, rigorous quality assurance, long-term storage stability, regulatory approval, and validation under realistic food-processing conditions. Addressing these challenges will be essential to bridge the gap between laboratory-scale demonstrations and practical deployment in antimicrobial food systems.

The recent application of safe-and-sustainable-by-design principles to LDPE food packaging containing multicomponent nanomaterials demonstrates how early-stage safety, processing, performance, and regulatory considerations can be integrated during material development [29]. Although this example was not based on catalytic nanozymes, the framework is highly relevant. For nanozyme-enabled food systems, premarket evaluation should combine antimicrobial performance with migration, worker exposure, consumer exposure, environmental release, and end-of-life analysis. This broader framework would help avoid a common translational failure: developing a material with excellent antimicrobial activity but insufficient evidence for food-contact safety, manufacturability, or regulatory acceptance.

### 6.3. Data-Driven Optimization and Future Translation Pathways

The next stage of catalytic nanozyme development will likely depend on data-driven design. Nanozyme performance is controlled by a high-dimensional set of variables, including elemental composition, particle size, morphology, crystal structure, defect density, surface charge, ligands, coordination environment, pH, temperature, substrate concentration, and matrix composition. Traditional trial-and-error synthesis cannot efficiently explore this design space. Machine learning has already been applied to predict and design nanozymes with desired catalytic activity, including explainable models that identify relationships between material descriptors and enzyme-like behavior [9]. Graphdiyne-based nanozyme discovery, high-throughput screening of bimetallic nanoparticles, and AI-assisted platforms for nanozyme activity prediction further demonstrate the potential of computational acceleration [96].

For antimicrobial food systems, however, machine learning must move beyond predicting catalytic constants in simplified buffer systems. The most useful models should link material descriptors to application-level outputs such as antimicrobial efficacy, biofilm reduction, migration potential, food matrix compatibility, sensory impact, cytotoxicity, cost, and environmental persistence [97]. This requires curated datasets that include negative results, standardized assay conditions, and comparable descriptors. At present, nanozyme datasets remain fragmented because different studies report different substrates, pH values, temperatures, bacterial strains, exposure times, and catalytic metrics [98]. Without harmonized reporting, predictive models may identify correlations that do not generalize to real food systems.

A practical data-driven framework should include three levels [99]. The first level is material informatics, where composition, active-site structure, surface chemistry, and synthesis conditions are linked to catalytic activity. The second level is biointerface informatics, where nanozyme properties are connected to bacterial adhesion, membrane interaction, biofilm penetration, and antimicrobial outcome. The third level is translation informatics, where safety, migration, manufacturability, regulatory constraints, and sustainability indicators are incorporated into multi-objective optimization. Such a framework would allow researchers to screen candidate nanozymes not only for maximum activity but for balanced performance across efficacy, safety, cost, and scalability. For antimicrobial food applications, machine learning should optimize both antibacterial efficacy and food-contact safety rather than catalytic activity alone [100]. Because food nanozymes must balance multiple objectives, including pathogen inactivation, low nanoparticle migration, controlled metal-ion release, and minimal cytotoxicity, multi-objective optimization offers a practical design strategy. Pareto frontier analysis can identify candidate nanozymes that achieve an optimal balance between antimicrobial performance and migration risk without unnecessarily sacrificing either objective. In addition, multi-task learning enables the simultaneous prediction of antibacterial activity, catalytic efficiency, migration behavior, and toxicity from shared material descriptors. Integrating these approaches with safe-by-design principles could accelerate the development of nanozymes that are both highly effective and suitable for practical food-contact applications.

## 7. Conclusions

Catalytic nanozymes represent an emerging class of engineered nanomaterials with considerable potential to advance antimicrobial strategies in food systems. Their enzyme-mimicking activities, structural tunability, environmental stability, and capacity for localized catalytic reactions provide important advantages over conventional antimicrobial agents and natural enzymes. By catalyzing reactive oxygen species generation, disrupting microbial membranes, weakening extracellular polymeric substances, and supporting pathogen detection or food preservation, nanozymes offer a multifunctional platform for addressing persistent challenges associated with foodborne pathogens, biofilms, food-contact surface contamination, and postharvest microbial spoilage.

A central message of this review is that the antimicrobial performance of nanozymes cannot be understood solely from material composition or reported inhibition efficiency. Instead, their function is governed by complex structure–activity relationships involving active-site chemistry, particle size, morphology, crystal phase, exposed facets, defect density, porosity, surface charge, heterostructure interfaces, and single-atom coordination environments. These structural features determine catalytic pathways, substrate activation, reactive species generation, bacterial interaction, and biofilm penetration. Therefore, future development should move beyond empirical screening toward rational nanozyme design guided by mechanistic understanding and standardized performance evaluation.

Despite promising progress, several barriers remain before catalytic nanozymes can be broadly translated into antimicrobial food technologies. Many studies still rely on simplified bacterial models, buffer-based catalytic assays, or short-term antimicrobial tests that do not fully represent real food matrices, mixed microbial communities, organic loads, storage conditions, or repeated-use food-contact environments. In addition, the same catalytic properties that make nanozymes effective antimicrobial agents may raise concerns regarding oxidative damage, nanoparticle migration, metal ion release, environmental persistence, and unintended effects on human or ecological systems. Thus, antimicrobial efficacy must be evaluated together with exposure, safety, stability, migration, degradation, and end-of-life behavior.

Safe-by-design principles should become a core framework for the next generation of antimicrobial nanozyme research. This approach requires the selection of lower-risk material components, controlled immobilization, reduced unnecessary release, food-compatible carriers, scalable fabrication, and early integration of toxicological and regulatory assessment. For packaging, coatings, and food-contact surfaces, immobilized or matrix-confined nanozymes may be more realistic than freely dispersed nanoparticles because they can localize catalytic activity while reducing potential migration. For edible coatings or direct food applications, more stringent evaluation of ingestion safety, gastrointestinal transformation, and microbiome interactions will be essential.

Industrial translation will also require greater standardization. Future studies should report not only antimicrobial reduction values but also catalytic kinetics, active-site stability, food matrix effects, reusability, migration behavior, sensory impact, cost, and compatibility with existing processing or packaging technologies. Data-driven approaches, including machine learning, high-throughput screening, explainable artificial intelligence, and multi-objective optimization, may accelerate the discovery of nanozymes that balance catalytic activity, antimicrobial efficacy, safety, manufacturability, and sustainability. However, these tools will only be useful if supported by high-quality, standardized, and application-relevant datasets.

Overall, catalytic nanozymes provide a compelling platform for developing next-generation antimicrobial food systems, but their future impact will depend on responsible engineering rather than activity enhancement alone. The most promising nanozyme technologies will be those that integrate strong structure–activity relationships, controlled catalytic function, food matrix compatibility, low exposure risk, scalable manufacturing, and regulatory readiness. By combining materials science, food microbiology, safety assessment, and data-driven design, catalytic nanozymes can move from laboratory demonstrations toward practical, sustainable, and safe antimicrobial solutions for modern food systems.

## Figures and Tables

**Table 1:** Representative nanozyme applications in antimicrobial food systems.

Application	Representative Nanozyme	Key Outcome
Pathogen detection	Pt/Zr-MOF	Rapid detection of *E. coli* O157 in food matrices via catalytic signal amplification [20]
Pathogen detection	Pt–Au bimetallic nanoparticles	>1000-fold higher sensitivity for *E. coli* O157:H7 lateral-flow detection [54]
Pathogen detection	Mannose-modified Prussian blue	Label-free LFIA with LOD of 10^2^ CFU/mL for *E. coli* O157:H7 [55]
Detection + disinfection	Multifunctional nanozyme platform	Simultaneous detection and elimination of *L. monocytogenes* [56]
Food preservation	Ce-MOF/chitosan coating	Antibacterial activity and extended strawberry and banana shelf life [24]
Food preservation	H_2_O_2_-responsive peptide nanozyme/alginate hydrogel	Combined antimicrobial action and moisture retention for postharvest preservation [25]
Antimicrobial therapy	Magnetic PDA/Fe_3_O_4_ nanozyme	ROS-enhanced antibacterial activity with localized retention and high biosafety [57]

**Table 2:** Representative nanozyme-enabled sensing platforms for rapid detection of foodborne pathogens and food contaminants.

Detection Platform	Representative Nanozyme	Target Bacteria	Detection Mechanism	Major Advantages	Representative Application
Colorimetric assay	Prussian blue nanoparticles	*E. coli* O157	Peroxidase-like catalytic color amplification	Label-free detection, high stability, simple visual readout	Foodborne pathogen screening [54]
Lateral flow immunoassay (LFIA)	Prussian blue nanozyme	*Salmonella typhimurium*, *Listeria monocytogenes*	Catalytic signal amplification replacing conventional labels	Portable, multiplex detection, rapid analysis	Milk quality monitoring [22]
Immunochromatographic assay	Au@Pt nanozyme	*Salmonella typhimurium*	Peroxidase-like enhancement of chromogenic signal	Higher sensitivity than AuNP labels	On-site food inspection [22]
Magnetic relaxation biosensor	Au nanoparticle-assisted system	Aflatoxin B1	Triple cascade amplification (DNA + HCR + nanoparticle)	Ultrasensitive toxin detection	Food contaminant monitoring [71]
Cascade catalytic biosensor	Single-atom Ce–N–C nanozyme	Organophosphate and carbamate pesticides	Peroxidase-like activity coupled with acetylcholinesterase	Portable detection within 30 min; high catalytic activity	Fruits and vegetables [72]
Microfluidic biosensor	Pt/Zr-MOF nanozyme	*E. coli* O157	Microfluidic immunoassay with catalytic amplification	Integrated sample preparation; rapid (<1 h); portable	Water, milk, cabbage [20]
Microfluidic immunosensor	Porous Au@Pt nanopompoms	*Salmonella typhimurium*	Filtration enrichment + nanozyme colorimetric amplification	Ultrasensitive detection with sample enrichment	Food samples [73]
SERS immunosensor	Bifunctional catalytic nanozyme	Foodborne pathogens	Nanozyme-assisted catalytic amplification combined with SERS	Extremely high sensitivity and specificity	Milk analysis [55]
Dual-mode sensor	TiO_2_–Fe–N–C nanozyme	*Listeria monocytogenes*	Photoelectrochemical + colorimetric detection	Cross-validation improves analytical accuracy	Food safety monitoring [74]
Theranostic nanozyme platform	Multifunctional nanozyme	*Listeria monocytogenes*	Simultaneous pathogen detection and antimicrobial action	Detect-and-eliminate capability	Smart antimicrobial food systems [74]

**Table 3:** Representative nanozyme-enabled active packaging and edible coating systems for food preservation.

Nanozyme System	Food Application	Key Functions and Advantages
Nanocellulose-based polymeric nanozyme spray coating	Fruit preservation	Bioinspired antimicrobial coating with enzyme-like catalytic activity to extend postharvest shelf life [80]
Chitosan/Ce-MOF nanozyme coating	Strawberries, bananas	Oxidase-like and apyrase-like activities suppress microbial growth and delay quality deterioration [81]
Biomineralized bimetallic MOF nanozyme packaging film	Fresh fruits	Catalytic nanozymes embedded within packaging films for sustained antimicrobial activity and preservation [82]
Tannic acid-chelated Ag nanozyme hydrogel	Antimicrobial food-contact materials	Self-setting adhesive hydrogel with peroxidase-like catalysis, ROS generation, Ag^+^ release, and enhanced mechanical stability [83]
Peptide nanozyme–alginate dual-network hydrogel	Strawberry preservation	H_2_O_2_-responsive antimicrobial coating that combines moisture retention with catalytic antibacterial activity while maintaining fruit quality [25,84]

## Data Availability

No new data were created or analyzed in this review study. Data sharing does not apply to this article.

## References

[1] Havelaar A.H., Kirk M.D., Torgerson P.R., Gibb H.J., Hald T., Lake R.J., Praet N., Bellinger D.C., de Silva N.R., Gargouri N. (2015). World Health Organization global estimates and regional comparisons of the burden of foodborne disease in 2010. PLoS Med..

[2] Likotrafiti E., Rhoades J., Watson R.R., Preedy V.R. (2016). Probiotics, prebiotics, synbiotics, and foodborne illness. Probiotics, Prebiotics, and Synbiotics: Bioactive Foods in Health Promotion.

[3] Abebe G.M. (2020). The role of bacterial biofilm in antibiotic resistance and food contamination. Int. J. Microbiol..

[4] Sorathiya K.B., Melo A., Hogg M.C., Pintado M. (2025). Organic acids in food preservation: Exploring synergies, molecular insights, and sustainable applications. Sustainability.

[5] Shi J., Xu J., Liu X., Goda A.A., Salem S.H., Deabes M.M., Ibrahim M.I.M., Naguib K., Mohamed S.R. (2024). Evaluation of some artificial food preservatives and natural plant extracts as antimicrobial agents for safety. Discov. Food.

[6] Nguyen H.L., Nguyen T.B.N. (2026). Evaluation of metal-doped ZIF-8-hyaluronic acid nanocomposites for disruption of *Salmonella Typhimurium* and *Escherichia coli* on food contact (stainless steel) surfaces. Sustainability.

[7] Nazir A., Xu X., Liu Y., Chen Y. (2023). Phage endolysins: Advances in the world of food safety. Cells.

[8] Gao L., Zhuang J., Nie L., Zhang J., Yu Z., Gu N., Wang T., Feng J., Yang D., Perrett S. (2007). Intrinsic peroxidase-like activity of ferromagnetic nanoparticles. Nat. Nanotechnol..

[9] Mu J., Wang Y., Zhao M., Zhang L. (2012). Intrinsic peroxidase-like activity and catalase-like activity of Co_3_O_4_ nanoparticles. Chem. Commun..

[10] Li J., Liu W., Wu X., Gao X. (2015). Mechanism of pH-switchable peroxidase and catalase-like activities of gold, silver, platinum and palladium. Biomaterials.

[11] Feng S., Wang Z., Zhang Y., Mei L., Wang Z. (2025). Harnessing nanozymes as next-generation antimicrobial agents: From mechanisms to therapeutic strategies. Mater. Today Bio.

[12] Vishwanath V., Ramanathan R., Neelakantan P. (2026). Nanozymes as antimicrobial agents in endodontics. JADA Found. Sci..

[13] Natalio F., André R., Hartog A.F., Stoll B., Jochum K.P., Wever R., Tremel W. (2012). Vanadium pentoxide nanoparticles mimic vanadium haloperoxidases and thwart biofilm formation. Nat. Nanotechnol..

[14] Liu Y., Naha P.C., Hwang G., Kim D., Huang Y., Simon-Soro A., Jung H.-I., Ren Z., Li Y., Gubara S. (2018). Topical ferumoxytol nanoparticles disrupt biofilms and prevent tooth decay *in vivo* via intrinsic catalytic activity. Nat. Commun..

[15] Gao F., Shao T., Yu Y., Xiong Y., Yang L. (2021). Surface-bound reactive oxygen species generating nanozymes for selective antibacterial action. Nat. Commun..

[16] Bilal M., Khaliq N., Ashraf M., Hussain N., Baqar Z., Zdarta J., Jesionowski T., Iqbal H.M.N. (2023). Enzyme mimic nanomaterials as nanozymes with catalytic attributes. Colloids Surf. B Biointerfaces.

[17] Wang X., Shi Q., Zha Z., Zhu D., Zheng L., Shi L., Wei X., Lian L., Wu K., Cheng L. (2021). Copper single-atom catalysts with photothermal performance and enhanced nanozyme activity for bacteria-infected wound therapy. Bioact. Mater..

[18] Feng Y., Qin J., Zhou Y., Yue Q., Wei J. (2022). Spherical mesoporous Fe–N–C single-atom nanozyme for photothermal and catalytic synergistic antibacterial therapy. J. Colloid Interface Sci..

[19] Li Z., Xu D., Deng Z., Yin J., Qian Y., Hou J.-T., Ding X., Shen J., He X. (2023). Single-atom-catalyzed MXene-based nanoplatform with photo-enhanced peroxidase-like activity nanotherapeutics for *Staphylococcus aureus* infection. Chem. Eng. J..

[20] Xing G., Shang Y., Ai J., Lin H., Wu Z., Zhang Q., Lin J.-M., Pu Q., Lin L. (2023). Nanozyme-mediated catalytic signal amplification for microfluidic biosensing of foodborne bacteria. Anal. Chem..

[21] Li Z., Hu J., Zhan Y., Shao Z., Gao M., Yao Q., Li Z., Sun S., Wang L. (2023). Coupling bifunctional nanozyme-mediated catalytic signal amplification and label-free SERS with immunoassays for ultrasensitive detection of pathogens in milk samples. Anal. Chem..

[22] Hendrickson O.D., Byzova N.A., Dzantiev B.B., Zherdev A.V. (2024). Prussian-blue-nanozyme-enhanced simultaneous immunochromatographic control of two relevant bacterial pathogens in milk. Foods.

[23] Li H., Ren Y., Zhan Y., Yu X., Zhang X., Zhu C., Ye Y. (2025). “Four-in-one” platform based on multifunctional nanozyme for ultra-accurate detection and on-demand disinfection of *Listeria monocytogenes*. Food Chem..

[24] Li J., Wang D., Liu Y. (2024). Biomimetic spray coating for fruit preservation based on UiO-167 metal–organic framework nanozyme. Front. Mater..

[25] Li X., Liao D., Zhou Q., Wang L., Sun L., Tong Z., Sun J., Zhang Z., Li D., Zhou G. (2026). H_2_O_2_-activatable peptide nanozyme-alginate dual-network hydrogel coating reconciling antibacterial and moisture retention in postharvest preservation. ACS Appl. Mater. Interfaces.

[26] Lin Q.-B., Li H., Zhong H.-N., Zhao Q., Xiao D.-H., Wang Z.-W. (2014). Migration of Ti from nano-TiO_2_-polyethylene composite packaging into food simulants. Food Addit. Contam. Part A Chem. Anal. Control Expo. Risk Assess..

[27] Cushen M., Kerry J., Morris M., Cruz-Romero M., Cummins E. (2014). Silver migration from nanosilver and a commercially available zeolite filler polyethylene composites to food simulants. Food Addit. Contam. Part A Chem. Anal. Control Expo. Risk Assess..

[28] Yang R., Liu Z., Chen H., Zhang X., Sun Q., El-Mesery H.S., Lu W., Dai X., Xu R. (2025). Advances in nanozyme catalysis for food safety detection: A comprehensive review on progress and challenges. Foods.

[29] Brunelli A., Trabucco S., Salgado C., Reinosa J.J., Fernandez J.F., Serrano-Lotina A., Bañares M.A., Blosi M., Peijnenburg W., Soeteman-Hernandez L.G. (2025). Safe and sustainable by design-compliant LDPE food packaging embedding multicomponent nanomaterials for food protection. Environ. Sci. Nano.

[30] Wei H., Wang E. (2008). Fe_3_O_4_ magnetic nanoparticles as peroxidase mimetics and their applications in H_2_O_2_ and glucose detection. Anal. Chem..

[31] Wang Y., Wu C., Sun X., Ye T., Liu C., Liu A., Wu X., Pang X. (2026). Beyond antibiotics: Engineered metal nanozymes for resistance-evading antibacterial therapy. J. Nanobiotechnol..

[32] Zhu Y., Yan J., Liu J., Chen H., Gui J., Wu C., Zhu X., Yin P., Liu M., Zhang Y. (2022). Multi-mimic activities of CO_3_O_4_ nanopolyhedrons and application in regulating the content of intracellular hydrogen peroxide/oxygen. ACS Appl. Nano Mater..

[33] Zhang R., Yan X., Gao L., Fan K. (2025). Nanozymes expanding the boundaries of biocatalysis. Nat. Commun..

[34] Gu H., Li J., Dai P., Sun T., Chen C., Guo Z., Fan K. (2025). Polyphenol oxidase-like nanozymes. Adv. Mater..

[35] Jomova K., Alomar S.Y., Alwasel S.H., Nepovimova E., Kuca K., Valko M. (2024). Several lines of antioxidant defense against oxidative stress: Antioxidant enzymes, nanomaterials with multiple enzyme-mimicking activities, and low-molecular-weight antioxidants. Arch. Toxicol..

[36] Filippova A.D., Sozarukova M.M., Baranchikov A.E., Kottsov S.Y., Cherednichenko K.A., Ivanov V.K. (2023). Peroxidase-like activity of CeO_2_ nanozymes: Particle size and chemical environment matter. Molecules.

[37] Jiang P., Zhang L., Liu X., Ye C., Zhu P., Tan T., Wang D., Wang Y. (2024). Tuning oxidant and antioxidant activities of ceria by anchoring copper single-site for antibacterial application. Nat. Commun..

[38] Burwell T., Thangamuthu M., Fernandes J.A., Khlobystov A.N. (2026). Mapping reaction pathways and catalyst dynamics in electrochemical CO_2_ reduction through in situ and *operando* characterisation. Chem. Commun..

[39] Yuan B., Tan Z., Guo Q., Shen X., Zhao C., Chen J.L., Peng Y.-K. (2023). Regulating the H_2_O_2_ activation pathway on a well-defined CeO_2_ nanozyme allows the entire steering of its specificity between associated enzymatic reactions. ACS Nano.

[40] Nguyen H.L., Nguyen T.B.N. (2026). Metal-doped and surface-functionalized ZIF-8 nanoplatforms for antimicrobial applications in food and environmental systems. Sustainability.

[41] Wei F., Cui X., Wang Z., Dong C., Li J., Han X. (2021). Recoverable peroxidase-like Fe_3_O_4_@MoS_2_-Ag nanozyme with enhanced antibacterial ability. Chem. Eng. J..

[42] Dai X., Liu H., Du W., Su J., Kong L., Ni S., Zhan J. (2023). Biocompatible carbon nitride quantum dots nanozymes with high nitrogen vacancies enhance peroxidase-like activity for broad-spectrum antibacterial. Nano Res..

[43] Feng F., Zhang Y., Zhang X., Mu B., Zhang J., Qu W., Tong W., Liang M., An Q., Guo Z. (2024). Enhancing the peroxidase-like activity of MoS_2_-based nanozymes by introducing attapulgite for antibacterial application and sensitive detection of glutathione. Nano Res..

[44] Jiang H., Xing Y., Ma Z., Fan G., Liu Z., Yang S., Cai L. (2026). Copper single-atom nanozyme with intelligent capture and photo-enhanced activity for controlling plant bacterial diseases. Nat. Commun..

[45] Xu W., Sun B., Wu F., Mohammadniaei M., Song Q., Han X., Wang W., Zhang M., Zhou N., Shen J. (2022). Manganese single-atom catalysts for catalytic-photothermal synergistic anti-infected therapy. Chem. Eng. J..

[46] Qu Y., Zhuang L., Bao W., Li C., Chen H., He S., Yao H., Si Q. (2024). Atomically dispersed nanozyme-based synergistic mild photothermal/nanocatalytic therapy for eradicating multidrug-resistant bacteria and accelerating infected wound healing. RSC Adv..

[47] Song Z., Xing C., Yuan Y., Liu B., Zhang X., Liu J., Huang Y., Han D., Zhang J. (2026). The bi-MOF@CQDs@g-C_3_N_4_ heterojunction photocatalyst enhances interface charge transfer via electron bridges to promote rapid norfloxacin degradation. Sep. Purif. Technol..

[48] Zhang M., Yue W., Ma W., Wang X., Xu Y., Li A. (2025). Heterostructure nanozyme with hyperthermia-amplified enzyme-like activity and controlled silver release for synergistic antibacterial therapy. Adv. Healthc. Mater..

[49] Sun H., Dan J., Liang Y., Li M., Zhuo J., Kang Y., Su Z., Zhang Q., Wang J., Zhang W. (2022). Dimensionality reduction boosts the peroxidase-like activity of bimetallic MOFs for enhanced multidrug-resistant bacteria eradication. Nanoscale.

[50] Nguyen H.L., Moreira R.G., Castell-Perez M.E. (2026). Multifunctional OEO-ZIF-8-HA nanoparticles for antibacterial control on latex surfaces and baby arugula (*Eruca sativa*) leaves. J. Food Sci..

[51] Georgakopoulos-Soares I., Papazoglou E.L., Karmiris-Obratański P., Karkalos N.E., Markopoulos A.P. (2023). Surface antibacterial properties enhanced through engineered textures and surface roughness: A review. Colloids Surf. B Biointerfaces.

[52] Cong Y., Qiao R., Wang X., Ji Y., Yang J., Baimanov D., Yu S., Cai R., Zhao Y., Wu X. (2024). Protein corona-mediated inhibition of nanozyme activity: Impact of protein shape. J. Am. Chem. Soc..

[53] Zhang X., Shi Y., Wu D., Fan L., Liu J., Wu Y., Li G. (2024). A bifunctional core-shell gold@Prussian blue nanozyme enabling dual-readout microfluidic immunoassay of food allergic protein. Food Chem..

[54] Jiang T., Song Y., Wei T., Li H., Du D., Zhu M.-J., Lin Y. (2016). Sensitive detection of *Escherichia coli* O157 using Pt-Au bimetal nanoparticles with peroxidase-like amplification. Biosens. Bioelectron..

[55] Wang Z., Yao X., Zhang Y., Wang R., Ji Y., Sun J., Zhang D., Wang J. (2020). Functional nanozyme-mediated multi-readout and label-free lateral flow immunoassay for rapid detection of *Escherichia coli* O157. Food Chem..

[56] Li M., Han T., Xia C., Xu J., Sun C., Chen J., Xu W., Wang D. (2025). A nanozyme-based dual-mode sensor for photoelectrochemical and colorimetric assay of *Listeria monocytogenes*. Food Chem..

[57] Xiao J., Hai L., Li Y., Li H., Gong M., Wang Z., Tang Z., Deng L., He D. (2022). An ultrasmall Fe_3_O_4_-decorated polydopamine hybrid nanozyme enables continuous conversion of oxygen into toxic hydroxyl radical via GSH-depleted cascade redox reactions for intensive wound disinfection. Small.

[58] Cao M., Ma J., Yang Y., Cheng M., Liu J., Pan Z., Du Z. (2026). Oxidoreductase-like nanozymes: From biosensing to molecular mechanisms in disease therapy. Int. J. Nanomed..

[59] Xu Y., Yan J., Cui C., Zhou L., Zhang K., Du M., Gong Y., Zhang Z., Wu X., Li B. (2025). Nanozymes empower periodontitis treatment: New strategies and clinical application prospects. Biomater. Res..

[60] Liu Y., Huang Y., Kim D., Ren Z., Oh M.J., Cormode D.P., Hara A.T., Zero D.T., Koo H. (2021). Ferumoxytol nanoparticles target biofilms causing tooth decay in the human mouth. Nano Lett..

[61] Qin Y., Qian C., Li W., Wang Q., Sheng Q., Chen Z., Zhang W., Li W., Ge G., Yan Z. (2026). Oxidative stress: Molecular mechanisms, diseases, and therapeutic targets. MedComm.

[62] Hu M., Korschelt K., Viel M., Wiesmann N., Kappl M., Brieger J., Landfester K., Thérien-Aubin H., Tremel W. (2018). Nanozymes in nanofibrous mats with haloperoxidase-like activity to combat biofouling. ACS Appl. Mater. Interfaces.

[63] Henych J., Ryšánek P., Šťastný M., Němečková Z., Adamec S., Kormunda M., Kamínková S., Hamalová K., Tolasz J., Janoš P. (2023). Electrospun PA6 nanofibers bearing the CeO_2_ dephosphorylation catalyst. ACS Omega.

[64] Chen Y.-S., Tian H.-X., Rong D.-C., Wang L., Chen S., Zeng J., Xu H., Mei J., Wang L.-Y., Liou Y.-L. (2025). ROS homeostasis in cell fate, pathophysiology, and therapeutic interventions. Mol. Biomed..

[65] Slavin Y.N., Asnis J., Häfeli U.O., Bach H. (2017). Metal nanoparticles: Understanding the mechanisms behind antibacterial activity. J. Nanobiotechnol..

[66] Wang Y., Feng Q., Liu M., Xue L., Wang G., Zhang S., Hu W. (2023). N, P, S codoped carbon nanozymes with enhanced peroxidase-like activity and binding affinity for total antioxidant capacity assay. ACS Appl. Nano Mater..

[67] Ding N., Dong S., Zhang Y., Lu D., Lin J., Zhao Q., Shi X. (2022). Portable silver-doped Prussian blue nanoparticle hydrogels for colorimetric and photothermal monitoring of shrimp and fish freshness. Sens. Actuators B Chem..

[68] Cai X., Huang Y., Zhu C. (2025). Immobilized multi-enzyme/nanozyme biomimetic cascade catalysis for biosensing applications. Adv. Healthc. Mater..

[69] Praveen N.G., Lalitha M.M., Sahoo J., Kurapati R. (2026). Biomimicking haloperoxidase by two-dimensional V_2_O_5_ nanosheets for marine antifouling applications. Chem. Asian J..

[70] Ma T., Huang K., Cheng N. (2023). Recent advances in nanozyme-mediated strategies for pathogen detection and control. Int. J. Mol. Sci..

[71] Hong F., Huang C., Wu L., Wang M., Chen Y., She Y. (2021). Highly sensitive magnetic relaxation sensing method for aflatoxin B_1_ detection based on Au NP-assisted triple self-assembly cascade signal amplification. Biosens. Bioelectron..

[72] Song G., Zhang J., Huang H., Wang X., He X., Luo Y., Li J.-C., Huang K., Cheng N. (2022). Single-atom Ce-N-C nanozyme bioactive paper with a 3D-printed platform for rapid detection of organophosphorus and carbamate pesticide residues. Food Chem..

[73] Cheng X., Chen H., Li W., Tu Z., Wang Y., Wei H., Wang S., Liu L., Rong Z. (2025). Nanozyme-catalyzed colorimetric microfluidic immunosensor for the filtration enrichment and ultrasensitive detection of *Salmonella Typhimurium* in food samples. Anal. Chem..

[74] Guo X., Chao M., Barimah A.O., Tai S., Ma W., Wang Z., Jin Z., Huang L., Peng C. (2026). Nanozyme-based “Detection-Plus” technology: Integrated detection and decontamination for food safety. Coord. Chem. Rev..

[75] Dong H., Du W., Dong J., Che R., Kong F., Cheng W., Ma M., Gu N., Zhang Y. (2022). Depletable peroxidase-like activity of Fe_3_O_4_ nanozymes accompanied with separate migration of electrons and iron ions. Nat. Commun..

[76] Shen H., Tang Y., Ma H. (2024). Accelerated peroxidase-like activity of ultrathin amine-tagged bimetallic MOF-74 nanozyme for construction of versatile bioassays from small molecules to enzymes and bacteria. Microchem. J..

[77] Huang Y., Liu Y., Pandey N.K., Shah S., Simon-Soro A., Hsu J.C., Ren Z., Xiang Z., Kim D., Ito T. (2023). Iron oxide nanozymes stabilize stannous fluoride for targeted biofilm killing and synergistic oral disease prevention. Nat. Commun..

[78] Chakraborty N., Gandhi S., Verma R., Roy I. (2022). Emerging prospects of nanozymes for antibacterial and anticancer applications. Biomedicines.

[79] Zhong H., Jiang C., Huang Y. (2023). The recent development of nanozymes for targeting antibacterial, anticancer and antioxidant applications. RSC Adv..

[80] Huang L., Sun D.-W., Pu H., Zhang C., Zhang D. (2023). Nanocellulose-based polymeric nanozyme as bioinspired spray coating for fruit preservation. Food Hydrocoll..

[81] Xu X., Zhang W., Jia S., Chen Y., Zhou X., Ding Y. (2025). Antimicrobial packaging films based on MOF nanozymes engineered via biomineralization and bimetallic hybridization for fruit preservation. Food Chem..

[82] Khan M.J., Hafeez F., Islam M.R., Zhu C., Xianyu Y. (2025). Advanced antibacterial packaging for food preservation through multifunctional metal–organic framework nanocomposite. Small.

[83] Jia Z., Lv X., Hou Y., Wang K., Ren F., Xu D., Wang Q., Fan K., Xie C., Lu X. (2021). Mussel-inspired nanozyme catalyzed conductive and self-setting hydrogel for adhesive and antibacterial bioelectronics. Bioact. Mater..

[84] He S., Ma L., Zheng Q., Wang Z., Chen W., Yu Z., Yan X., Fan K. (2024). Peptide nanozymes: An emerging direction for functional enzyme mimics. Bioact. Mater..

[85] Suvarna V., Nair A., Mallya R., Khan T., Omri A. (2022). Antimicrobial nanomaterials for food packaging. Antibiotics.

[86] Muzeza C., Ngole-Jeme V., Msagati T.A.M. (2023). The mechanisms of plastic food-packaging monomers’ migration into food matrix and the implications on human health. Foods.

[87] Bazilio F.S., dos Santos L.M.G., Silva C.B., Vicentini Neto S.A., Senna C.A., Archanjo B.S., Jacob S.C., Abrantes S.M.P. (2023). Migration of silver nanoparticles from plastic materials, with antimicrobial action, destined for food contact. J. Food Sci. Technol..

[88] Dokprom K., Sirisinha K., Wirasate S., Saenmuangchin R., Jimenez Lamana J., Bunchuay T., Siripinyanond A. (2025). Quantitative evaluation of silver nanoparticle migration from polyethylene-based packaging and their transformation in simulated gastrointestinal fluids by single particle ICP-MS. Microchem. J..

[89] Dekkers S., Adam V., Di Battista V., Haase A., Prinz J., Nagel G., Fransman W., Persson M., Suarez-Merino B., Wohlleben W. (2025). Safe-and-sustainable-by-design approach and decision support system for advanced materials. Adv. Sustain. Syst..

[90] U.S. Food and Drug Administration (2014). Considering Whether an FDA-Regulated Product Involves the Application of Nanotechnology: Guidance for Industry.

[91] EFSA Scientific Committee (2021). Guidance on risk assessment of nanomaterials to be applied in the food and feed chain: Human and animal health. EFSA J..

[92] Jiang B., Duan D., Gao L., Zhou M., Fan K., Tang Y., Xi J., Bi Y., Tong Z., Gao G.F. (2018). Standardized assays for determining the catalytic activity and kinetics of peroxidase-like nanozymes. Nat. Protoc..

[93] OECD (2023). Test No. 125: Nanomaterial Particle Size and Size Distribution of Nanomaterials. OECD Guidelines for the Testing of Chemicals, Section 1.

[94] Xia X., Liu X., Gao Y., Lin J., Pan S., Cheng W., Huang S., Liu X., Shen J.-W., Duan W. (2025). Engineered metallic hybrid nanozyme for advanced inflammatory disease therapy. Mater. Today Bio.

[95] Deng X., Cao S., Horn A.L. (2021). Emerging applications of machine learning in food safety. Annu. Rev. Food Sci. Technol..

[96] OECD (2017). Test No. 318: Dispersion Stability of Nanomaterials in Simulated Environmental Media. OECD Guidelines for the Testing of Chemicals, Section 3.

[97] Yu Y., Jiang Y., Zhang C., Bai Q., Fu F., Li S., Wang L., Yu W.W., Sui N., Zhu Z. (2022). Machine learning assisted graphdiyne-based nanozyme discovery. ACS Mater. Lett..

[98] Nguyen H.L., Nguyen H.M.X., Nguyen T.B.N. (2026). Data-driven engineering of antimicrobial nanomaterials for food safety and biomedical systems. Nanomaterials.

[99] Wei Y., Wu J., Wu Y., Liu H., Meng F., Liu Q., Midgley A.C., Zhang X., Qi T., Kang H. (2022). Prediction and design of nanozymes using explainable machine learning. Adv. Mater..

[100] Liu J., Wang Y., Xie Y., Zhang C., Zhang J., Chen L., Zhao L., Li B., Yang H., Yong Y. (2026). Programming nanozyme identity for precision catalytic medicine by tailoring the bio-nano interface. Int. J. Pharm..

